# Structural and Potentially Modifiable Anthropometric Traits in Professional Kenyan and European Long-Distance Runners: A Cross-Sectional Study

**DOI:** 10.3390/jfmk11030292

**Published:** 2026-07-26

**Authors:** Fabrizio Spataro, Camila Seewald, Jimena Moreira Lamas, Francis Holway, Francesco Campa

**Affiliations:** 1 PhD School of Applied Medical-Surgical Sciences, University of Rome “Tor Vergata”, 00133 Rome, Italy; fabrizio.spataro@gmail.com; 2 Department of Human Nutrition and Dietetics, Universitat de Carlemany, AD700 Escaldes-Engordany, Andorra; cbseewald@gmail.com (C.S.); jmoreiralamas@gmail.com (J.M.L.); 3 Department of Nutrition and Physical Activity, University of Barcelona, 08028 Barcelona, Spain; fholway@gmail.com; 4 Department of Biomedical Sciences, University of Padua, 35124 Padua, Italy

**Keywords:** anthropometry, body composition, body proportions, fat mass, ISAK, skinfolds

## Abstract

**Background:** East African runners, particularly from Kenya, have long dominated long-distance running, whereas highly trained European runners represent an appropriate comparison population to investigate structural and potentially modifiable anthropometric characteristics. **Objective:** To compare structural and modifiable anthropometric traits between professional Kenyan and European long-distance runners and to investigate the relationship between local muscularity and skeletal robustness. **Methods:** A total of 52 male marathon runners (32 Kenyan and 20 European) were assessed according to the International Society for the Advancement of Kinanthropometry (ISAK) protocol. Participants were 27.2 ± 5.2 years with a BMI of 18.4 ± 1.1 kg·m^−2^ and a marathon time of 02:09:26 in the Kenyan group, and 30.9 ± 4.9 years, BMI 21.1 ± 2.1 kg·m^−2^, and marathon time of 02:18:31 in the European group. Corrected girths were calculated to explore correlations between skeletal breadths and muscularity. **Results:** European runners exhibited significantly greater body mass, sitting height, girths, skeletal breadths, and muscle mass compared to Kenyan runners (*p* < 0.05), with large to very large effect sizes. In contrast, Kenyan runners showed lower adiposity and more linear body proportions, including a lower Cormic index and higher intermembral index. Differences in segment lengths were generally limited. Principal component analysis showed clearer group separation for modifiable than structural traits. Significant positive correlations were observed between humeral breadth and corrected arm girth (r = 0.41, *p* = 0.002), femur breadth and corrected thigh girth (r = 0.66, *p* < 0.001), and bimalleolar breadth and corrected calf girth (r = 0.72, *p* < 0.001). **Conclusions:** These findings suggest that Kenyan long-distance runners exhibit a lighter and more linear anthropometric profile than their European counterparts, whereas European runners display greater skeletal robustness and musculoskeletal development. Furthermore, local muscularity may be partly associated with underlying skeletal robustness.

## 1. Introduction

East African runners, particularly from Kenya, have dominated middle- and long-distance running for decades [1]. This phenomenon has been partly attributed to superior running economy [2,3,4], which is considered one of the primary physiological determinants of endurance performance beyond maximal oxygen uptake alone [5,6]. Previous studies have suggested that these characteristics are associated with improved running economy, potentially through reductions in lower-limb moment of inertia and enhanced mechanical efficiency during running [7,8].

However, the interpretation of lower local muscularity in Kenyan runners remains incomplete. Muscle-related traits are often considered predominantly modifiable characteristics influenced by training and nutritional status. Nevertheless, recent magnetic resonance imaging-based evidence has demonstrated that skeletal dimensions are among the strongest predictors of muscularity, suggesting a close coupling between bone size and muscle volume [9]. These findings raise the possibility that the lower muscularity observed in Kenyan runners may not solely reflect training-related or modifiable factors, but could also be partially associated with lower skeletal robustness and a more gracile musculoskeletal structure [6].

In this context, anthropometric approaches based on skeletal breadths and adiposity-corrected girths may provide useful field-based proxies to investigate the relationship between structural robustness and local muscularity [10]. This may be particularly relevant in endurance athletes, who are generally not exposed to hypertrophy-oriented training stimuli [7]. Furthermore, distinguishing between structural and modifiable traits may improve the understanding of the morphological phenotype associated with elite endurance running [5].

Although the anthropometric characteristics of Kenyan endurance runners have been extensively investigated, fewer studies have directly compared these athletes with highly trained European long-distance runners using standardized anthropometric methodologies. European runners represent an appropriate comparison because they are exposed to similar endurance training demands while differing in genetic background, developmental history, and environmental influences. Consequently, comparing these populations may help distinguish predominantly structural anthropometric characteristics from those that are more susceptible to training and environmental adaptations. Furthermore, although previous studies have described the body size and proportions of elite endurance runners [8,11], the relationship between skeletal robustness and local muscularity has received little attention, particularly using practical field-based anthropometric methods. Addressing this knowledge gap may contribute to a better understanding of the structural and modifiable components of the endurance athlete phenotype.

Based on these considerations, the aim of the present study was to compare structural and modifiable anthropometric traits between Kenyan and European long-distance runners and to investigate whether local muscularity was associated with skeletal robustness through the analysis of skeletal breadths and corrected girths. We hypothesized that Kenyan runners would exhibit a more linear and less robust musculoskeletal phenotype characterized by smaller skeletal breadths, lower corrected girths, and reduced body mass components compared with European runners. In addition, we hypothesized that skeletal breadths would be positively associated with adiposity-corrected girths, particularly in the lower limbs, suggesting that local muscularity may be partly related to underlying skeletal robustness.

## 2. Materials and Methods

Before participation, all subjects were informed about the study procedures and provided written informed consent. The study protocol was approved by the Ethics Committee of the University of Padua on 31 July 2023 (approval code: HECDSB.022023) and conducted in accordance with the Declaration of Helsinki. The study is reported in accordance with the STROBE recommendations for cross-sectional studies [12].

### 2.1. Participants

This cross-sectional study was conducted at the athletes’ training centers between December 2024 and January 2026. Eligible participants were male marathon runners competing at the national or international level who were actively engaged in structured endurance training. Athletes with injuries or medical conditions affecting anthropometric assessment, or with incomplete anthropometric data, were excluded. The Kenyan group (32 athletes) consisted predominantly of athletes from the Kalenjin ethnic group, including Kipsigis, Nandi, Marakwet, and Keiyo subgroups, whereas the European group (20 athletes) comprised runners from several European countries, including Italy, Spain, the Netherlands, Norway, Germany, Denmark, and Slovenia. All anthropometric assessments were performed under standardized conditions approximately 10–12 weeks before competition.

### 2.2. Measurement Procedures

**Figure 1:**
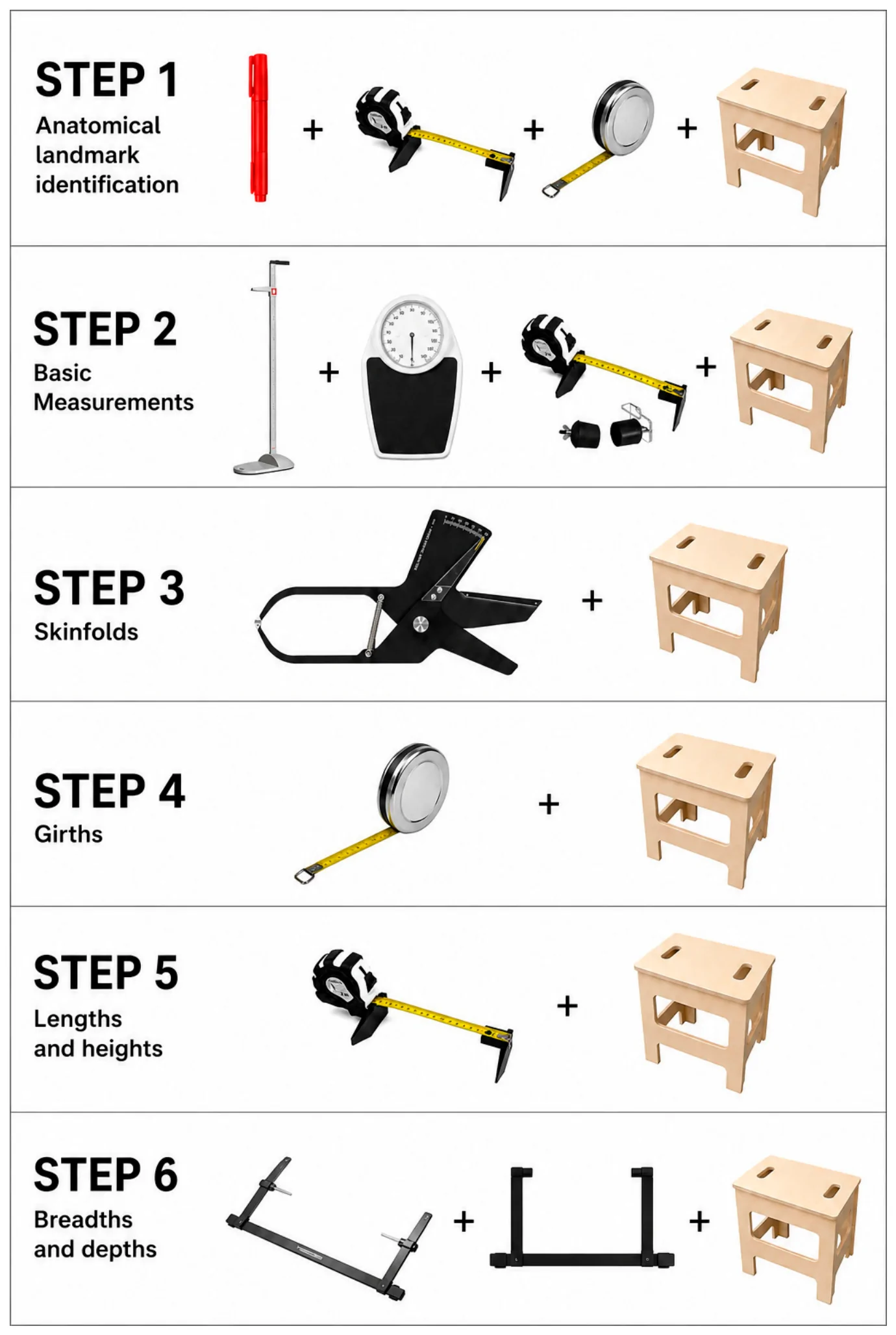
Anthropometric equipment used during the assessment according to the ISAK protocol. The figure illustrates the instruments used for anatomical landmark identification, basic anthropometric measurements, skinfold thicknesses, girths, segment lengths and heights, and skeletal breadths and depths.

Anthropometric assessments were conducted by operators (Level 2 and 3) certified by the International Society for the Advancement of Kinanthropometry (ISAK), following standardized international procedures [13]. An overview of the anthropometric equipment used throughout the standardized ISAK assessment protocol is presented in Figure 1. ISAK certification requires verification of intra- and inter-tester measurement precision according to internationally standardized criteria. Intra-operator Technical Error of Measurement (TEM) values were below 5% for skinfold thicknesses and below 1% for all other anthropometric measurements. Before data collection, anatomical landmarks were identified and marked according to the standardized ISAK protocol using an anthropometric marker, anthropometric tape, and anthropometric box where appropriate. Body mass and stature were measured using a scale with an integrated stadiometer (Seca, Hamburg, Germany) to the nearest 0.1 kg and 0.1 cm, respectively. Sitting height was measured using the same stadiometer in combination with an anthropometric box, whereas arm span was measured using an anthropometer. Skinfold thicknesses were measured using a type A [14] skinfold caliper (Holway, Berkeley, CA, USA) at eight anatomical sites: triceps, subscapular, biceps, iliac crest, supraspinale, abdominal, thigh, and calf, and their sum was calculated. Girth measurements were obtained using a non-elastic anthropometric tape (Lufkin, Apex Tool Group, Huntersville, NC, USA) with a sensitivity of 0.1 mm, including head, neck, arm relaxed, flexed and tensed arm, forearm, wrist, chest, waist, hips, thigh 1 cm gluteal, thigh middle, calf, and ankle. Segment lengths and skeletal breadths were measured to the nearest 0.1 cm using anthropometric calipers (Holway, Berkeley, CA, USA), including sliding and breadth calipers according to the anatomical site. These included biacromial, biliocristal, chest, humerus, femur, bi-styloid, and bimalleolar breadths, as well as multiple segment lengths (upper and lower limbs and trunk-related measures).

### 2.3. Body Composition Assessment

Muscle mass, bone mass, and the muscle-to-bone ratio were estimated using anthropometric prediction equations based on the Kerr fractionation model [15], based on the proportionality system relative to the Phantom reference developed by Drinkwater and Ross [16]. For each tissue compartment (muscle and bone), a Z-score was calculated using the equation Z = [(V × (170.18/H)) − P]/s, where V is the sum of the compartment-specific anthropometric variables, H is stature (cm), P is the Phantom reference value, and s is the corresponding Phantom standard deviation. In the equation M = (Z × s + P) × (H/170.18)^3^, M represents the estimated tissue mass expressed in kilograms, Z is the standardized Phantom Z-score, s is the Phantom standard deviation, P is the Phantom reference value, and H is stature in centimetres. Muscle mass was estimated from the sum of corrected girths (arm, forearm, chest, thigh, and calf), adjusted for subcutaneous adipose tissue using the equation: Corrected girths = C − π × (SKF/10), where C is girth (cm) and SKF is the corresponding skinfold thickness in mm, and π is the mathematical constant pi. Bone mass was estimated by combining the contribution of the body (based on biacromial and biliocristal breadths, and humerus and femur bicondylar breadths) and the head (derived from head girth), following the original Kerr formulation. Tissue masses were calculated independently of total body mass, and no post hoc adjustment was applied to match measured body mass. Fat mass was estimated using an anthropometric equation developed using Hologic dual-energy X-ray absorptiometry as the reference method and based on the eight skinfold thicknesses included in the standardized ISAK protocol [17]. Somatotype was determined according to the Heath–Carter anthropometric method [18]. Several anthropometric proportional indices were derived from the linear and breadth measurements to characterize segmental proportions and overall body shape. The relative arm span was calculated as arm span divided by stature and multiplied by 100, whereas the Cormic index was calculated as sitting height divided by stature and multiplied by 100. These indices were used to describe upper-limb reach and trunk proportionality relative to total body height, respectively. The brachial index was calculated as radiale–stylion length divided by acromiale–radiale length and multiplied by 100, thereby expressing forearm length relative to upper arm length. The intermembral index was calculated as total upper-limb length divided by total lower-limb length and multiplied by 100, where total upper-limb length was defined as the sum of acromiale–radiale, radiale–stylion, and midstylion–dactylion lengths, and total lower-limb length as the iliospinale height. The crural index was calculated as tibiale mediale-sphyrion tibiale length divided by trochanterion–tibiale laterale length and multiplied by 100, representing lower leg length relative to thigh length. Finally, the acromio-iliac index was calculated as biliocristal breadth divided by biacromial breadth and multiplied by 100 and was used to describe shoulder-to-pelvis proportionality. All indices were expressed as percentages.

### 2.4. Statistical Analysis

All statistical analyses were performed using RStudio.2026.07.1 Data are presented as mean ± standard deviation (SD). Normality of distributions was assessed using the Shapiro–Wilk test, and homogeneity of variances was evaluated using Levene’s test. Between-group differences (Kenyan vs. European runners) were assessed using independent samples *t*-tests. In cases where the assumption of equal variances was violated, Welch’s *t*-test was applied. All tests were two-tailed. To control for multiple comparisons, *p*-values were adjusted using the Benjamini–Hochberg false discovery rate (FDR) procedure. Effect sizes were calculated using Hedges’ g and interpreted as small (0.2), moderate (0.5), and large (0.8), with values > 1.2 considered very large [19]. To assess the potential confounding effect of age on the between-group comparisons, additional analyses of covariance (ANCOVA) were performed for all anthropometric variables, with group (Kenyan vs. European) as the fixed factor and age as a continuous covariate. The assumption of homogeneity of regression slopes was verified by testing the Group × Age interaction before fitting the final models. Adjusted *p*-values are reported as p_FDR throughout the manuscript. Principal component analysis (PCA) was performed on standardized variables (z-scores) based on the correlation matrix. PCA was used as an exploratory dimensionality reduction technique for visualization of clustering patterns. Two separate PCAs were conducted to distinguish between (i) predominantly structural traits (including stature, skeletal breadths, and proportional indices) and (ii) modifiable traits (including body mass, girths, skinfolds, and body composition variables). The number of components retained was based on explained variance, and the first two principal components were used for visualization. To further investigate the relationship between skeletal robustness and local muscularity, corrected girths were calculated according to the ISAK methodology by subtracting the adipose tissue component from the corresponding limb girths using the equation: corrected girth = girth − π × (skinfold/10), where girth was expressed in centimetres and skinfold thickness in millimetres. Pearson correlation analyses were performed between skeletal breadths and corrected girths, including humeral breadth and corrected arm girth, femur breadth and corrected thigh girth, and bimalleolar breadth and corrected calf girth. A sensitivity analysis was performed to determine the minimum detectable standardized effect size based on the available sample (32 Kenyan and 20 European runners), assuming a two-sided significance level of α = 0.05 and 80% statistical power. The available sample size was sufficient to detect standardized between-group effect sizes of Cohen’s d ≥ 0.81. Statistical significance was set at *p* < 0.05.

## 3. Results

### 3.1. General Characteristics and Body Size

The Kenyan runners were aged 27.2 ± 5.2 years, had a body mass index (BMI) of 18.4 ± 1.1 kg·m^−2^, and a mean marathon time of 02:09:26. The European runners were aged 30.9 ± 4.9 years, had a BMI of 21.1 ± 2.1 kg·m^−2^, and a mean marathon time of 02:18:31. Descriptive statistics and between-group comparisons are reported in Table 1, Table 2, Table 3, Table 4, Table 5, Table 6 and Table 7. European runners exhibited significantly higher values in body mass and sitting height compared to Kenyan runners (both *p* < 0.05), with large effect sizes (Table 1). Stature was also greater in European runners, although with a smaller effect size.

Additional ANCOVA adjusting for age confirmed the observed between-group differences in body mass (F = 34.16, p_FDR < 0.001), stature (F = 6.61, p_FDR = 0.013), BMI (F = 28.13, p_FDR < 0.001), and sitting height (F = 37.43, p_FDR < 0.001).

### 3.2. Girths and Skeletal Breadths

European runners showed significantly higher values in all major girth measurements, including chest, waist, hip, and limb girths (all *p* < 0.05; Table 2), with very large effect sizes (Hedges’ g often > 1.5). The largest differences were observed for hip and waist girths. Similarly, skeletal breadths and body depths (Table 3) were generally greater in European runners, particularly for biacromial breadth, transverse chest, and biliocristal breadth (all *p* < 0.05). However, the magnitude of these differences was smaller compared to girths.

Additional ANCOVA adjusting for age confirmed the observed between-group differences in head (F = 21.96, p_FDR < 0.001), neck (F = 7.28, p_FDR = 0.011), relaxed arm (F = 24.14, p_FDR < 0.001), flexed and tensed arm (F = 23.42, p_FDR < 0.001), forearm (F = 6.15, p_FDR = 0.018), chest (F = 30.54, p_FDR < 0.001), waist (F = 29.40, p_FDR < 0.001), hip (F = 51.97, p_FDR < 0.001), thigh 1 cm gluteal (F = 24.71, p_FDR < 0.001), thigh middle (F = 23.69, p_FDR < 0.001), calf (F = 20.64, p_FDR < 0.001), and ankle girths (F = 16.56, p_FDR < 0.001), whereas wrist girth remained non-significant (F = 1.04, p_FDR = 0.313).

Additional ANCOVA adjusting for age confirmed the observed between-group differences in biacromial breadth (F = 6.45, p_FDR = 0.026), antero-posterior abdominal depth (F = 13.88, p_FDR = 0.002), transverse chest breadth (F = 11.45, p_FDR = 0.004), biliocristal breadth (F = 17.69, p_FDR < 0.001), femur breadth (F = 8.94, p_FDR = 0.010), and bimalleolar breadth (F = 5.11, p_FDR = 0.042). In contrast, antero-posterior chest depth (F = 0.30, p_FDR = 0.721), humerus breadth (F = 0.04, p_FDR = 0.846), and bi-styloid breadth (F = 0.22, p_FDR = 0.721) remained non-significant after age adjustment.

### 3.3. Segment Lengths and Heights

Differences in segment lengths and heights were less pronounced (Table 4). Kenyan runners showed higher values in selected upper-limb segment lengths, whereas European runners exhibited greater foot length (*p* < 0.05). Most other segmental variables did not differ significantly between groups after FDR correction.

Additional ANCOVA adjusting for age confirmed the observed between-group differences in acromiale–radiale length (F = 12.44, p_FDR = 0.009), radiale–stylion length (F = 6.39, p_FDR = 0.049), and foot length (F = 6.96, p_FDR = 0.049). Arm span (F = 2.16, p_FDR = 0.246), midstylion–dactylion length (F = 1.92, p_FDR = 0.246), iliospinale height (F = 0.11, p_FDR = 0.931), trochanterion height (F < 0.01, p_FDR = 0.959), trochanterion–tibiale laterale length (F = 2.54, p_FDR = 0.235), tibiale laterale height (F = 4.55, p_FDR = 0.095), and tibiale mediale–sphyrion tibiale length (F = 0.02, p_FDR = 0.959) remained non-significant after age adjustment.

### 3.4. Skinfolds

European runners presented higher values in several skinfold measurements and in the sum of eight skinfolds (Table 5), although not all comparisons remained significant after FDR correction. Kenyan runners consistently showed lower adiposity values.

Additional ANCOVA adjusting for age confirmed the observed between-group differences in triceps (F = 6.06, p_FDR = 0.039), supraspinale (F = 6.14, p_FDR = 0.039), thigh (F = 12.94, p_FDR = 0.007), and the sum of eight skinfolds (F = 7.65, p_FDR = 0.036). Subscapular (F = 1.95, p_FDR = 0.190), biceps (F = 0.27, p_FDR = 0.603), iliac crest (F = 2.02, p_FDR = 0.190), abdominal (F = 3.12, p_FDR = 0.125), and calf skinfolds (F = 3.62, p_FDR = 0.113) remained non-significant after age adjustment.

### 3.5. Body Mass Components and Somatotype

**Figure 2:**
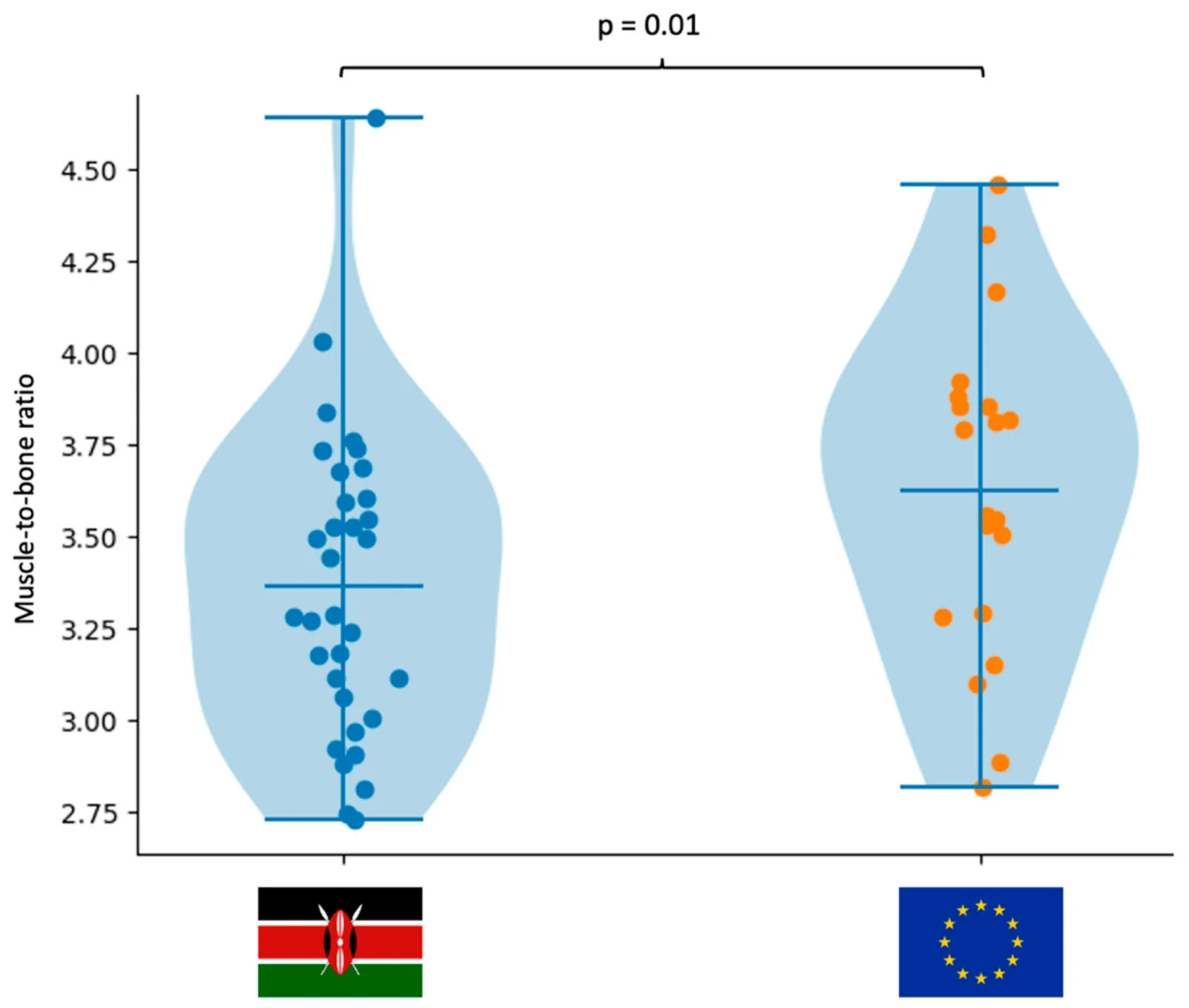
Muscle-to-bone ratio of the Kenyan and European runners.

Analysis of body mass components (Table 6) revealed that European runners had significantly greater SMM, bone mass, FM, FMI, and SMI (all *p* < 0.05), with large effect sizes. Although the muscle-to-bone ratio was also significantly higher in European runners (*p* < 0.05) (Figure 2), the effect size was moderate, indicating limited biological relevance compared to absolute differences in SMM and bone mass.

Additional ANCOVA adjusting for age confirmed the observed between-group differences in bone mass (F = 17.41, p_FDR < 0.001), muscle mass (F = 30.32, p_FDR < 0.001), muscle-to-bone ratio (F = 5.25, p_FDR = 0.031), fat mass (F = 12.02, p_FDR = 0.002), FMI (F = 9.74, p_FDR = 0.004), SMI (F = 21.67, p_FDR < 0.001), endomorphy (F = 5.12, p_FDR = 0.031), mesomorphy (F = 9.71, p_FDR = 0.004), and ectomorphy (F = 18.18, p_FDR < 0.001). In contrast, fat mass percentage remained non-significant after age adjustment (F = 0.45, p_FDR = 0.506).

**Figure 3:**
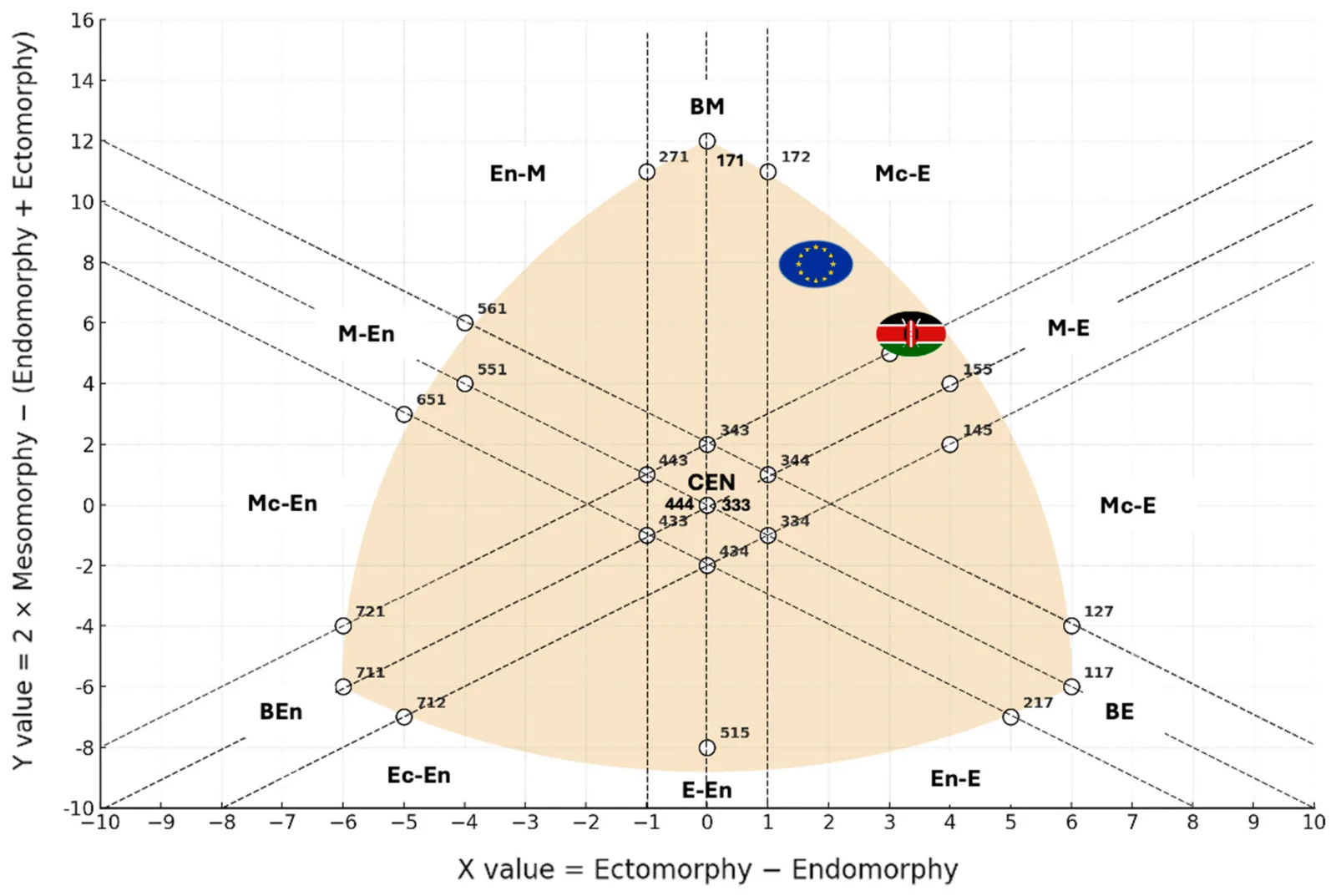
Somatochart with the main coordinates used to define the 13 somatotype categories. The somatotypes of the Kenyan and European runners are plotted with their respective centroids. CEN (Central), BE (Balanced Ectomorph), BEn (Balanced Endomorph), BM (Balanced Mesomorph), E-En (Ectomorph–Endomorph), Ec-En (Ectomorphic–Endomorph), Ec-M (Ectomorphic–Mesomorph), En-E (Endomorphic–Ectomorph), En-M (Endomorphic–Mesomorph), M-E (Mesomorph–Ectomorph), Mc-E (Mesomorphic–Ectomorph), M-En (Mesomorph–Endomorph), and Mc-En (Mesomorphic–Endomorph).

The somatotype analysis illustrated in Figure 3 showed that both Kenyan and European runners were distributed within the mesomorphic–ectomorphic region, with minor differences in somatotype components.

### 3.6. Proportional Indices

**Figure 4:**
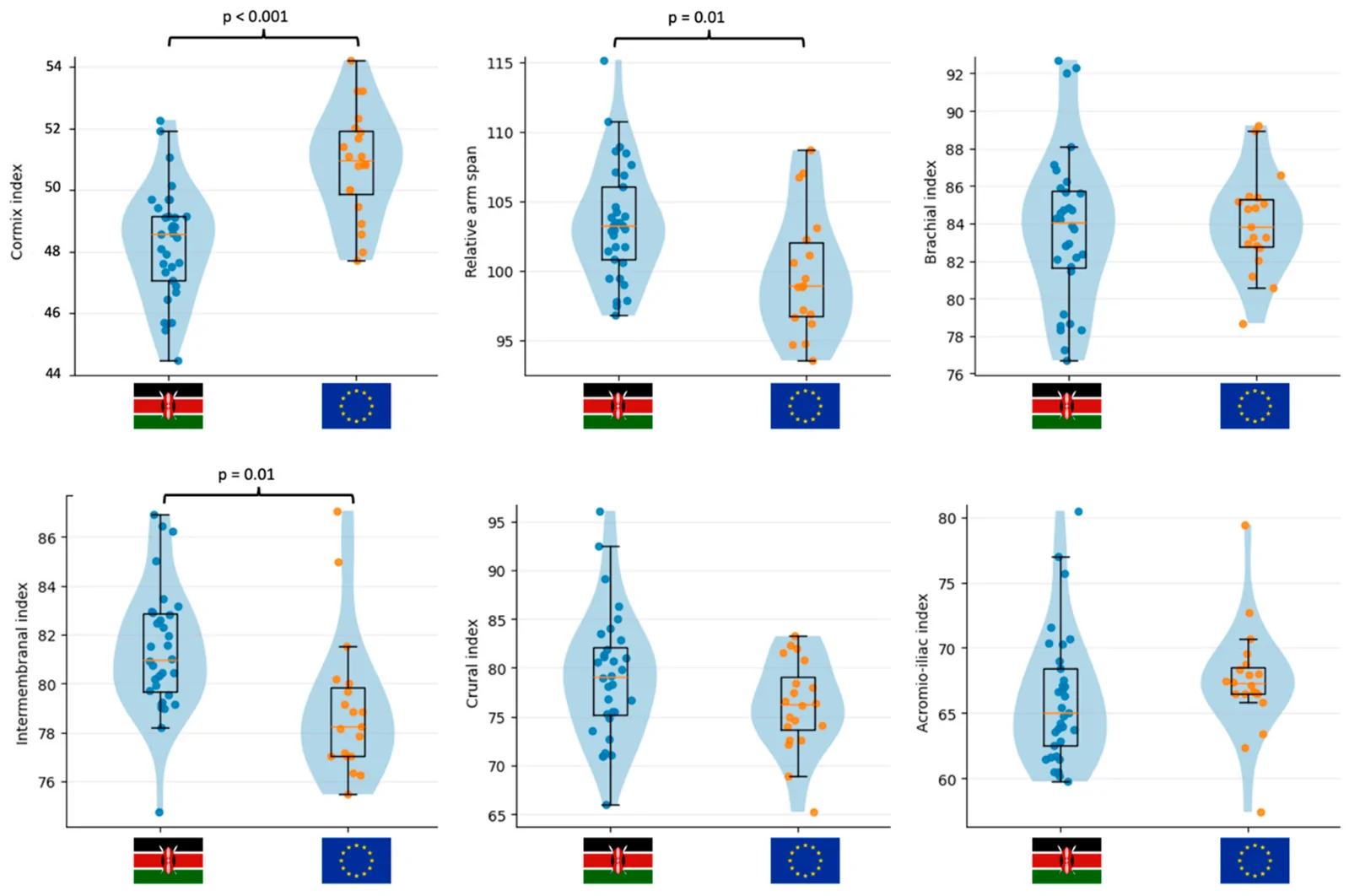
Violin plots showing the distribution of selected proportional indices in the Kenyan and European runners.

Analysis of proportional indices (Table 7 and Figure 4) revealed distinct morphological patterns between groups. Kenyan runners exhibited significantly lower Cormic index values (*p* < 0.001), indicating relatively shorter trunk length in relation to stature, and significantly higher relative arm span and intermembral index (*p* < 0.05), reflecting relatively longer limb segments. Differences in brachial, crural, and acromio-iliac indices were smaller and did not consistently reach statistical significance after correction.

Additional ANCOVA adjusting for age confirmed the observed between-group differences in Cormic index (F = 22.28, p_FDR < 0.001), relative arm span (F = 6.72, p_FDR = 0.025), and intermembral index (F = 7.88, p_FDR = 0.021). In contrast, brachial index (F = 0.06, p_FDR = 0.808), crural index (F = 2.48, p_FDR = 0.183), and acromio-iliac index (F = 1.73, p_FDR = 0.233) remained non-significant after age adjustment.

### 3.7. Principal Component Analysis

**Figure 5:**
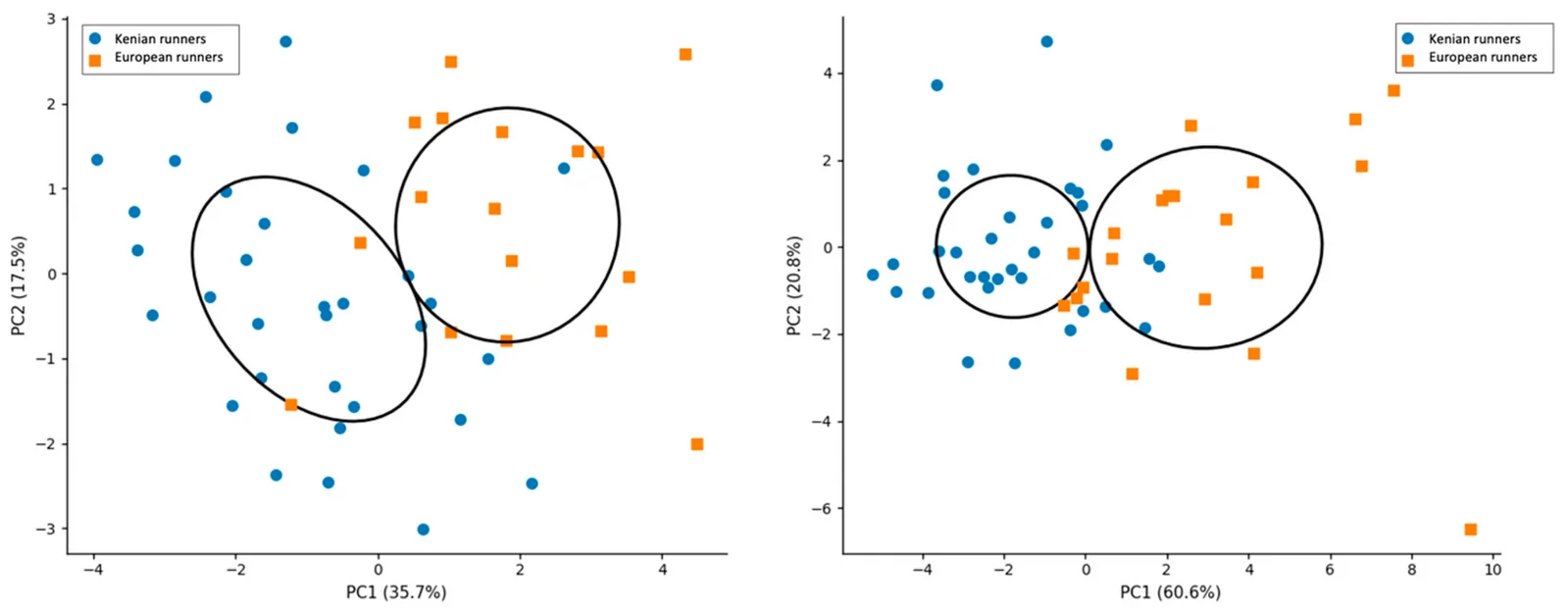
Principal component analysis (PCA) of predominantly structural traits and modifiable traits in Kenyan and European runners. Percentages on axes indicate explained variance for each principal component.

PCA was performed on standardized variables (z-scores) separately for predominantly structural and modifiable traits, as shown in Figure 5, respectively. The predominantly structural dataset included stature, sitting height, arm span, biacromial breadth, biliocristal breadth, femur breadth, bimalleolar breadth, cormic index, relative arm span, brachial index, intermembral index, crural index, and acromio-iliac index. The modifiable dataset included body mass, chest, waist, hip, thigh middle, and calf girth, sum of 8 skinfolds, bone mass, SMM, muscle-to-bone ratio, FM, FMI, SMI, endomorphy, mesomorphy, and ectomorphy. These variables represent body composition, adiposity, muscularity, and somatotype components, which are more susceptible to training and environmental influences.

In the PCA of predominantly structural traits, the first principal component (PC1) explained 35.7% of the total variance and was mainly associated with overall body size and skeletal breadths (e.g., stature, biacromial and femur breadths), whereas the second component (PC2; 17.5%) was primarily driven by proportional indices (e.g., Cormic index, relative arm span, and intermembral index). Kenyan and European runners showed partial separation along PC1, although substantial overlap remained between groups. In contrast, the PCA of modifiable traits showed a clearer separation between groups. PC1 explained 60.6% of the variance and was strongly associated with body mass, girths (chest, waist, hip), and SMM, representing a dimension primarily associated with body size and muscularity-related variables. The second component (PC2; 20.8%) was more closely related to adiposity variables (skinfolds, FMI, and endomorphy). Kenyan runners were predominantly distributed at lower PC1 scores, whereas European runners clustered at higher values, indicating greater body mass, girths, and overall musculoskeletal development. No collinearity filtering was applied because the purpose of PCA was dimensionality reduction and pattern visualization rather than predictive variable selection.

### 3.8. Correlations Between Skeletal Breadths and Corrected Girths

**Figure 6:**
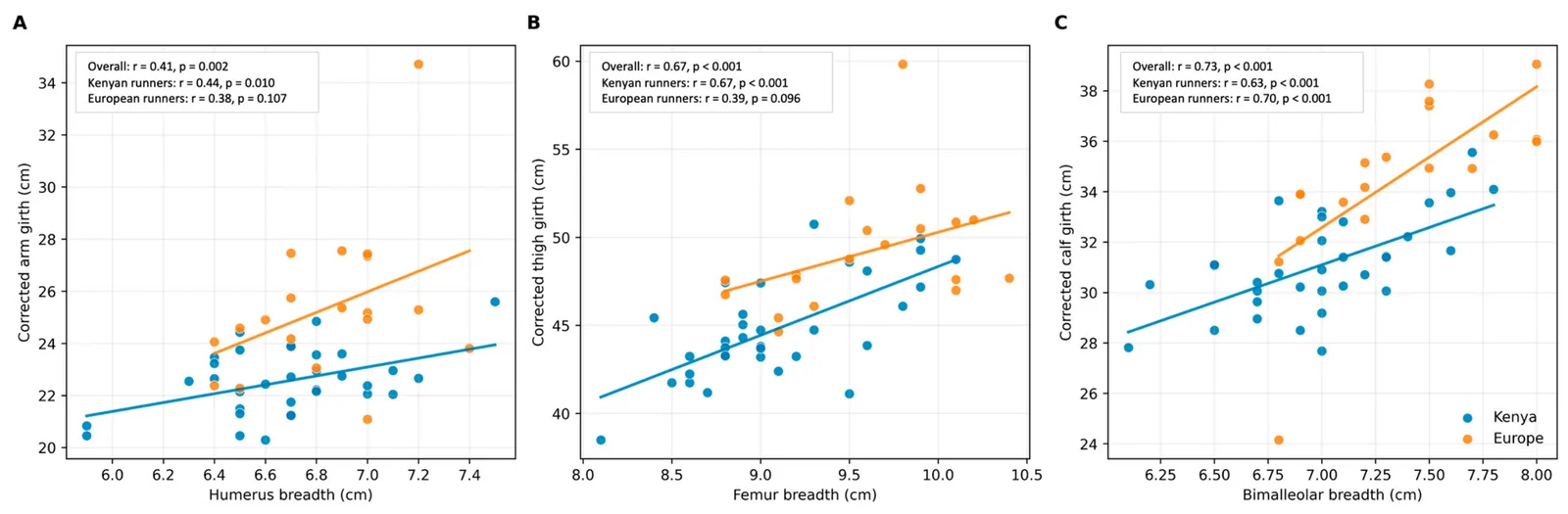
Pearson correlations between skeletal breadths and corrected girths in Kenyan and European marathon runners. Scatterplots illustrate the relationships between humerus breadth and corrected arm girth (**A**), femur breadth and corrected thigh girth (**B**), and bimalleolar breadth and corrected calf girth (**C**). Kenyan and European runners are displayed separately, with group-specific linear regression lines. Pearson correlation coefficients are reported for the overall sample and separately for each group.

To investigate whether local muscularity was related to skeletal robustness, corrected girths were calculated by adjusting limb girths for the corresponding skinfold thicknesses. Significant positive correlations were observed between humeral breadth and corrected arm girth (r = 0.41, *p* = 0.002), femur breadth and corrected thigh girth (r = 0.67, *p* < 0.001), and bimalleolar breadth and corrected calf girth (r = 0.73, *p* < 0.001), as shown in Figure 6. Additional within-group analyses demonstrated that the positive correlations were generally maintained in the Kenyan runners, including humeral breadth and corrected arm girth (r = 0.44, *p* = 0.010), femur breadth and corrected thigh girth (r = 0.67, *p* < 0.001), and bimalleolar breadth and corrected calf girth (r = 0.63, *p* < 0.001). In the European runners, a significant positive correlation was observed only between bimalleolar breadth and corrected calf girth (r = 0.70, *p* < 0.001), whereas positive but non-significant trends were found for humeral breadth and corrected arm girth (r = 0.38, *p* = 0.107) and femur breadth and corrected thigh girth (r = 0.39, *p* = 0.096).

## 4. Discussion

The primary aim of the present study was to compare structural anthropometric traits and modifiable body composition characteristics between Kenyan and European long-distance runners and to investigate whether local muscularity was associated with skeletal robustness. The European runners exhibited greater body mass, girths, skeletal breadths, and muscle-related body composition variables, whereas the Kenyan runners displayed a more linear body configuration characterized by lower Cormic index values and relatively longer limb proportions. In addition, significant positive correlations were observed between skeletal breadths and adiposity-corrected girths, particularly in the lower limbs, suggesting that local muscularity may be associated with underlying skeletal robustness.

The present findings are consistent with previous studies describing East African endurance athletes as having lower body mass, slimmer lower limbs, and relatively longer limb proportions compared with non-African runners [8,20]. In particular, the Kenyan runners exhibited lower girths, smaller skeletal breadths, and lower body mass components related to muscularity, whereas proportional indices reflected a more linear morphology. Specifically, the lower Cormic index observed in the Kenyan runners indicates a relatively shorter trunk in relation to stature, which indirectly reflects relatively longer lower limbs. In contrast, the higher relative arm span and intermembral index values suggest proportionally longer upper limbs relative to stature and lower-limb length. These proportional characteristics are consistent with the anthropometric profile previously described in elite Kenyan endurance runners [8,20], although their functional implications cannot be inferred from the present study because running economy and biomechanical variables were not directly assessed.

The European runners exhibited a significantly higher muscle-to-bone ratio compared with Kenyan runners, indicating a greater amount of skeletal muscle mass relative to bone mass and reflecting a generally more robust musculoskeletal phenotype. However, the subsequent correlations observed between skeletal breadths and corrected girths suggest that local muscularity may still be partially associated with underlying skeletal robustness. These findings are consistent with recent MRI-based evidence demonstrating strong muscle–bone coupling across multiple body regions, where skeletal dimensions were identified as major predictors of muscle volume (“big bones mean big muscles”) [9]. Interestingly, despite the large between-group differences observed for absolute muscle and bone mass, the muscle-to-bone ratio showed only a moderate effect size. This finding suggests that the proportional relationship between skeletal and muscular tissues was relatively preserved, whereas the absolute scale of both compartments differed substantially between Kenyan and European runners. Because the present study did not include physiological or biomechanical measurements, no conclusions can be drawn regarding the functional implications of this proportional relationship. Nevertheless, these findings support the interpretation that the observed differences primarily reflect overall musculoskeletal size rather than major alterations in the balance between muscle and skeletal tissue.

Although the present study used anthropometric proxies rather than direct imaging techniques, the observed relationships between skeletal breadths and corrected girths support the concept that local muscularity may not exclusively reflect lower muscle mass. This interpretation may also help explain previous observations reporting lower lower-limb volumes and slimmer lower legs in Kenyan runners [8]. In this context, the reduced limb girths observed in the Kenyan runners may not exclusively reflect lower muscle mass, but may also be partially associated with a more gracile skeletal structure. Conversely, the higher muscle-to-bone ratio observed in the European runners may also be influenced by differences in skeletal robustness, but could also be indirectly related to their greater skeletal mass and overall structural robustness. Comparisons with previous studies investigating muscle-to-bone relationships should nevertheless be interpreted cautiously because different methodological approaches may capture distinct biological compartments [21]. For example, some investigations have calculated muscle-to-bone ratio using dual X-ray absorptiometry-derived lean soft mass and bone mineral content, which represent molecular-level body composition variables [22], whereas the present study estimated skeletal muscle mass and bone mass at the tissue level using anthropometric equations derived from cadaver-based models [23,24,25]. Consequently, these indices are not directly interchangeable and may reflect different aspects of musculoskeletal morphology. Overall, these findings suggest that muscle-related anthropometric characteristics may also be associated with underlying skeletal robustness rather than being exclusively explained by training-related adaptations.

These findings are also consistent with previous evidence in elite Kenyan marathon runners reporting a predominantly ectomorphic somatotype, characterized by low endomorphy and mesomorphy combined with a highly linear body configuration [26]. In that study, the Kenyan runners exhibited very low body mass, reduced adiposity, and slender segmental morphology, features considered potentially advantageous for running economy and long-distance performance. One of the most relevant findings of the present study was that several variables traditionally considered modifiable, particularly muscle-related girths and SMI, also appeared to show correlations with structural characteristics. Significant positive correlations were observed between skeletal breadths and adiposity-corrected girths, especially in the lower limbs, indicating that local muscularity may be partially associated with skeletal robustness. The strength of these correlations progressively increased from the upper to the lower limbs, with the strongest relationships observed between femur breadth and corrected thigh girth and between bimalleolar breadth and corrected calf girth. Interestingly, the PCA of predominantly structural traits suggested that skeletal robustness and body proportionality may represent partially independent morphological dimensions, whereas the PCA of modifiable variables was predominantly driven by body mass, girths, and skeletal muscle mass. These findings are consistent with anthropometric and imaging-based evidence suggesting that skeletal dimensions are closely associated with local muscularity and body composition characteristics [10,27]. In endurance athletes, this interaction may be particularly relevant in the lower limbs, where reduced skeletal robustness and slimmer distal segments have been associated with improved running economy and lower energetic cost of locomotion [6,8]. Accordingly, part of the variability in local muscularity observed among endurance runners may reflect interactions between structural morphology and training-related adaptations.

The stronger correlations observed in the lower limbs compared with the upper limbs may reflect the functional specialization of these segments in endurance running. Lower-limb morphology plays a central role in running economy, force transmission, and elastic energy utilization during locomotion [28,29]. The additional within-group analyses further supported this interpretation, showing that the positive correlations between femur breadth and corrected thigh girth, as well as between bimalleolar breadth and corrected calf girth, were also observed within both Kenyan and European runners, although statistical significance was not consistently reached in the European group, likely reflecting the smaller sample size. In contrast, the relationship between humeral breadth and corrected arm girth reached statistical significance only in the Kenyan runners, whereas a positive but non-significant trend was observed in the European runners. These findings suggest that the relationship between skeletal robustness and local muscularity is more consistent in the lower limbs than in the upper limbs, while also indicating that the pooled correlations were not solely driven by the overall differences between Kenyan and European runners. Therefore, the combination of lower skeletal robustness and reduced local muscularity may contribute to the slender lower-limb morphology commonly observed in Kenyan endurance runners. Importantly, lower skeletal breadths should not be interpreted as reduced skeletal adaptation or impaired bone health. Previous studies have reported high proximal femur bone mineral density in elite Kenyan runners despite their generally slender morphology, likely reflecting adaptation to repetitive endurance loading and high training volumes [2]. Therefore, skeletal geometry and skeletal density should be considered distinct aspects of musculoskeletal adaptation. Kenyan runners may exhibit a more gracile skeletal morphology while simultaneously maintaining high functional adaptation of bone tissue.

The present findings suggest that field anthropometry may provide useful information regarding the interaction between skeletal structure and local muscularity in endurance runners. From a practical perspective, the present findings suggest that anthropometric assessment based on the ISAK protocol may help coaches and sport scientists distinguish between predominantly structural characteristics and traits that are potentially modifiable through training and nutritional interventions. Such information may improve the interpretation of body composition profiles in elite endurance runners and support individualized athlete monitoring without relying on imaging techniques. However, several limitations should be acknowledged. First, the study relied on anthropometric proxies rather than imaging-derived measurements of muscle and bone volumes; therefore, the estimated body composition variables should not be interpreted as direct measurements. Moreover, the anthropometric equations used to estimate body composition were not specifically developed or validated in elite long-distance runners. Nevertheless, to our knowledge, no anthropometric prediction equations specifically designed for this population are currently available [30]. Therefore, equations based on the standardized ISAK protocol were selected as the most appropriate available option for estimating body composition in the present study [15,17]. Second, the cross-sectional design precludes any inference regarding the direction or causality of the observed relationships and does not allow discrimination between genetic, developmental, environmental, and training-related contributors to the observed morphological differences. Third, although all participants competed at the national or international marathon level, the study was not designed to compare performance-matched athletes. Therefore, residual confounding related to differences in competitive performance cannot be completely excluded. Furthermore, the smaller European sample may have limited the statistical power to detect moderate within-group correlations, particularly for upper-limb variables. Fourth, the sample included only male runners, limiting the generalizability of the findings to female athletes and other endurance disciplines. Finally, direct measures of running economy or performance-related physiological variables were not available, and training volume, nutritional practices, altitude exposure, and other environmental factors were not controlled. Therefore, any potential functional or performance-related implications of the observed anthropometric characteristics remain speculative and should be confirmed by future studies integrating anthropometric, physiological, biomechanical, and imaging-based assessments.

## 5. Conclusions

Professional Kenyan long-distance runners exhibited a lighter and more linear anthropometric profile than their European counterparts, whereas European runners displayed greater skeletal robustness, muscularity, and overall body mass. Differences in limb lengths were relatively limited, while body proportions and body composition contributed more substantially to the distinct morphological profiles observed between groups. Furthermore, the positive correlations between skeletal breadths and corrected girths suggest that local muscularity may be partly associated with underlying skeletal robustness, particularly in the lower limbs. Overall, these findings support the usefulness of standardized field anthropometry for distinguishing predominantly structural from potentially modifiable anthropometric traits in elite endurance runners and may contribute to a more informed interpretation of body composition profiles in both research and applied sports settings. Future longitudinal studies incorporating imaging techniques and performance-related physiological measurements are warranted to further clarify the relative contribution of structural, environmental, and training-related factors to the anthropometric phenotype of elite endurance athletes.

## Figures and Tables

**Table 1:** General characteristics of Kenyan and European runners.

Variable	Kenyan Runners (Mean ± SD)	European Runners (Mean ± SD)	p_FDR	Hedges g
Age (years)	27.23 ± 5.17	30.85 ± 4.90	0.011	−0.81
Body mass (kg)	55.00 ± 4.88	65.30 ± 6.10	<0.001	−1.93
Stature (cm)	172.78 ± 5.32	178.90 ± 6.40	0.003	−0.91
Body mass index (kg·m^−2^)	18.40 ± 1.13	21.09 ± 2.06	<0.001	−1.71
Sitting height (cm)	83.38 ± 3.70	89.55 ± 4.20	<0.001	−1.96

Values are presented as mean ± standard deviation (SD); *p*-values were adjusted for multiple comparisons using the Benjamini–Hochberg false discovery rate (FDR) procedure. Effect sizes were calculated using Hedges’ g and interpreted as small (0.2), moderate (0.5), and large (0.8), with values > 1.2 considered very large.

**Table 2:** Girth measurements in the Kenyan and European runners.

Variable	Kenya (Mean ± SD)	Europe (Mean ± SD)	p_FDR	Hedges g
Head (cm)	54.62 ± 1.50	56.80 ± 1.80	<0.001	−1.57
Neck (cm)	33.56 ± 1.90	36.40 ± 2.20	0.001	−1.10
Arm relaxed (cm)	23.76 ± 1.80	27.30 ± 2.10	<0.001	−1.63
Arm flexed and tensed (cm)	26.83 ± 2.10	30.40 ± 2.50	<0.001	−1.72
Forearm (cm)	23.54 ± 1.50	25.80 ± 1.90	0.003	−1.06
Wrist (cm)	15.04 ± 0.90	15.70 ± 1.10	0.090	−0.52
Chest (cm)	84.05 ± 4.80	92.70 ± 6.10	<0.001	−1.85
Waist (cm)	69.27 ± 4.50	77.80 ± 5.80	<0.001	−1.93
Hip (cm)	82.86 ± 4.20	91.15 ± 5.30	<0.001	−2.36
Thigh 1 cm gluteal (cm)	48.18 ± 3.20	54.60 ± 3.90	<0.001	−1.66
Thigh (cm)	46.31 ± 2.90	52.10 ± 3.40	<0.001	−1.58
Calf (cm)	32.08 ± 2.10	35.10 ± 2.50	<0.001	−2.00
Ankle (cm)	19.63 ± 1.20	21.80 ± 1.40	<0.001	−1.48

Values are presented as mean ± standard deviation (SD); *p*-values were adjusted for multiple comparisons using the Benjamini–Hochberg false discovery rate (FDR) procedure. Effect sizes were calculated using Hedges’ g and interpreted as small (0.2), moderate (0.5), and large (0.8), with values > 1.2 considered very large.

**Table 3:** Skeletal breadths and depths in the Kenyan and European runners.

Variable	Kenyan Runners (Mean ± SD)	European Runners (Mean ± SD)	p_FDR	Hedges g
Biacromial (cm)	39.21 ± 2.10	42.50 ± 2.80	0.003	−0.97
Transverse chest (cm)	26.62 ± 1.50	29.10 ± 2.10	<0.001	−1.49
Antero-posterior chest (cm)	19.30 ± 1.10	19.80 ± 1.30	0.386	−0.23
Antero-posterior abdominal (cm)	17.95 ± 1.80	21.30 ± 2.40	<0.001	−1.32
Biliocristal (cm)	25.70 ± 1.90	28.10 ± 2.10	<0.001	−1.39
Humerus (cm)	6.68 ± 0.50	7.10 ± 0.60	0.096	−0.50
Bi-styloid (cm)	5.49 ± 0.40	5.80 ± 0.50	0.229	−0.43
Femur (cm)	9.09 ± 0.70	10.20 ± 0.80	0.001	−1.04
Bimalleolar (cm)	7.00 ± 0.60	7.90 ± 0.70	0.003	−0.90

Values are presented as mean ± standard deviation (SD); *p*-values were adjusted for multiple comparisons using the Benjamini–Hochberg false discovery rate (FDR) procedure. Effect sizes were calculated using Hedges’ g and interpreted as small (0.2), moderate (0.5), and large (0.8), with values > 1.2 considered very large.

**Table 4:** Segment lengths and heights in the Kenyan and European runners.

Variable	Kenya (Mean ± SD)	Europe (Mean ± SD)	p_FDR	Hedges g
Arm span (cm)	178.61 ± 6.50	180.90 ± 7.20	0.315	0.39
Acromiale-radiale (cm)	33.68 ± 1.80	31.80 ± 2.00	0.018	0.99
Radiale-stylion (cm)	28.19 ± 1.50	27.20 ± 1.70	0.065	0.70
Midstylion-dactylion (cm)	20.05 ± 1.10	19.30 ± 1.20	0.165	0.53
Iliospinale height (cm)	100.62 ± 4.20	100.80 ± 4.50	0.897	−0.04
Trochanterion height (cm)	100.58 ± 4.30	101.10 ± 4.70	0.827	−0.09
Troch-tibiale laterale (cm)	51.43 ± 3.10	49.90 ± 3.40	0.069	−0.57
Tibiale laterale height (cm)	49.60 ± 3.00	50.80 ± 3.30	0.315	0.38
Tibiale mediale-sphyrion tibiale (cm)	40.77 ± 2.80	40.50 ± 2.90	0.827	0.10
Foot (cm)	25.60 ± 1.20	27.10 ± 1.50	0.015	−0.86

Values are presented as mean ± standard deviation (SD); *p*-values were adjusted for multiple comparisons using the Benjamini–Hochberg false discovery rate (FDR) procedure. Effect sizes were calculated using Hedges’ g and interpreted as small (0.2), moderate (0.5), and large (0.8), with values > 1.2 considered very large.

**Table 5:** Skinfold thickness in the Kenyan and European runners.

Variable	Kenya (Mean ± SD)	Europe (Mean ± SD)	p_FDR	Hedges g
Triceps (mm)	3.72 ± 0.92	4.90 ± 1.20	0.020	−0.92
Subscapular (mm)	5.97 ± 1.13	6.90 ± 1.50	0.108	−0.49
Biceps (mm)	2.40 ± 0.40	2.30 ± 0.50	0.445	0.24
Iliac crest (mm)	5.52 ± 1.65	6.80 ± 2.00	0.097	−0.60
Supraspinale (mm)	3.95 ± 0.88	5.10 ± 1.20	0.014	−0.91
Abdominal (mm)	6.19 ± 2.26	7.80 ± 2.90	0.061	−0.71
Thigh (mm)	4.75 ± 1.03	6.80 ± 1.50	0.014	−1.21
Calf (mm)	3.14 ± 0.49	3.80 ± 0.70	0.097	−0.62
Σ8SKF (mm)	35.87 ± 7.39	44.50 ± 9.10	0.014	−0.97

Values are presented as mean ± standard deviation (SD); *p*-values were adjusted for multiple comparisons using the Benjamini–Hochberg false discovery rate (FDR) procedure. Effect sizes were calculated using Hedges’ g and interpreted as small (0.2), moderate (0.5), and large (0.8), with values > 1.2 considered very large. Σ8SKF, sum of eight skinfolds thicknesses.

**Table 6:** Body composition estimates in the Kenyan and European runners.

Variable	Kenya (Mean ± SD)	Europe (Mean ± SD)	p_FDR	Hedges g
Bone Mass (kg)	6.95 ± 1.02	8.10 ± 1.20	<0.001	−1.46
Muscle Mass (kg)	23.21 ± 3.38	27.90 ± 3.90	<0.001	−1.81
Muscle-to-bone ratio	3.33 ± 0.35	3.60 ± 0.40	0.016	−0.77
Fat mass (kg)	7.07 ± 1.33	9.40 ± 2.10	<0.001	−1.29
Fat mass (%)	12.83 ± 2.08	13.90 ± 2.40	0.131	−0.46
FMI (kg/m^2^)	2.36 ± 0.39	3.10 ± 0.60	<0.001	−1.20
SMI (kg/m^2^)	7.77 ± 1.02	9.10 ± 1.30	<0.001	−1.69
Endomorphy	1.09 ± 0.31	1.60 ± 0.50	0.006	−0.93
Mesomorphy	5.43 ± 0.89	6.30 ± 1.00	<0.001	−1.03
Ectomorphy	4.71 ± 0.75	3.20 ± 0.80	<0.001	1.39

Values are presented as mean ± standard deviation (SD); *p*-values were adjusted for multiple comparisons using the Benjamini–Hochberg false discovery rate (FDR) procedure. Effect sizes were calculated using Hedges’ g and interpreted as small (0.2), moderate (0.5), and large (0.8), with values > 1.2 considered very large. MBR, muscle to bone ratio; FMI, fat mass index; SMI, skeletal muscle mass index.

**Table 7:** Proportional indices in the Kenyan and European runners.

Variable	Kenya (Mean ± SD)	Europe (Mean ± SD)	p_FDR	Hedges g
Cormic index	48.3 ± 1.80	52.0 ± 2.00	<0.001	−1.45
Relative arm span	103.49 ± 4.13	100.10 ± 3.90	0.012	0.88
Brachial index	83.75 ± 4.13	84.20 ± 4.50	0.764	−0.08
Intermembral index	81.37 ± 2.57	79.00 ± 2.80	0.012	0.88
Crural index	79.11 ± 6.50	76.90 ± 5.80	0.086	0.51
Acromio-iliac index	66.12 ± 4.99	67.50 ± 5.20	0.364	−0.28

Values are presented as mean ± standard deviation (SD); *p*-values were adjusted for multiple comparisons using the Benjamini–Hochberg false discovery rate (FDR) procedure. Effect sizes were calculated using Hedges’ g and interpreted as small (0.2), moderate (0.5), and large (0.8), with values > 1.2 considered very large.

## Data Availability

The data presented in this study are available on reasonable request from the corresponding author.
